# Evaluation of Exclusion Netting for Coffee Berry Borer (*Hypothenemus Hampei*) Management

**DOI:** 10.3390/insects11060364

**Published:** 2020-06-11

**Authors:** Melissa A. Johnson, Samuel Fortna, Nicholas C. Manoukis

**Affiliations:** 1 Daniel K. Inouye US Pacific Basin Agricultural Research Center, United States Department of Agriculture–Agricultural Research Service, Hilo, HI 96720, USA; sam.fortna@usda.gov (S.F.); nicholas.manoukis@usda.gov (N.C.M.); 2 Oak Ridge Institute for Science and Education, Oak Ridge Associated Universities, Oak Ridge, TN 37830, USA; 3 Department of Plant and Environmental Protection Sciences, College of Tropical Agriculture and Human Resources, University of Hawaii at Manoa, Honolulu, HI 96822, USA

**Keywords:** *coffea arabica*, Hawaii, insect netting, integrated pest management, shade coffee, sustainable agriculture, physical barriers

## Abstract

Exclusion nets are increasingly being used to protect a variety of agricultural crops from insect pests as a sustainable alternative to chemical controls. We examined the efficacy of exclusion nets in controlling the world’s most damaging insect pest of coffee, *Hypothenemus hampei* (coffee berry borer), on two small-scale coffee farms on Hawai’i Island. We recorded microclimate data, fruit infestation, population per fruit, sex ratio, mortality by *Beauveria bassiana*, coffee yield and quality in four paired exclusion and control (un-netted) plots on both farms. Mean and maximum daily temperature and relative humidity were similar between treatments, while mean and maximum daily solar radiation was reduced by ~50% in exclusion plots. Green and ripe fruit from exclusion plots had significantly lower infestation compared to un-netted control plots at both farms. We observed no significant difference between exclusion and control plots in the number of CBB per fruit or the female:male sex ratio. CBB mortality was significantly higher in control relative to exclusion plots in one of the two farms. Ripe fruits harvested from exclusion plots were on average significantly heavier and wider than those from control plots; however, there was no significant difference in the average yield per tree between treatments. Lastly, coffee quality was not significantly different between control and exclusion plots. Our results suggest that with complete sanitation prior to net installation in an environment where CBB is actively circulating, exclusion netting can successfully control CBB on small-scale coffee farms without reducing coffee yield or quality, and has the potential to lower production and labor costs by eliminating the need to spray pesticides.

## 1. Introduction

The most damaging insect pest of coffee worldwide, Coffee Berry Borer (CBB), *Hypothenemus hampei* Ferrari (Coleoptera: Curculionidae: Scolytinae), is a tiny beetle ~2 mm in length which spends the majority of its life cycle in the seed of the coffee fruit. The adult female CBB bores into the coffee fruit through the central disc, and eventually into the seed itself, where she builds galleries to lay her eggs. The offspring develop inside the seed and feed on the endosperm tissue [1], reducing both coffee yield and quality [2]. Mature male and female siblings mate inside their natal fruit, after which the males die [3,4]. The mated females leave their natal fruit and fly in search of a new fruit to colonize and deposit their eggs [3,4]. A female can lay as many as 119 eggs in a single fruit [5], with individual fruits able to support multiple generations of CBB [6]. The number of generations per year varies depending on development temperatures, with two generations per season reported in Ethiopia, three in Kenya, and four in Colombia and Tanzania [7].

Synthetic insecticides such as endosulfan and chlorpyrifos have been used extensively in many countries to control CBB [8,9]. However, the toxicity of these insecticides to humans, wildlife and the environment [10,11,12,13,14], as well as evidence of insecticide resistance [15,16], have resulted in these substances being banned in an increasing number of countries [17,18,19]. Additionally, because CBB are only vulnerable to chemical controls during the narrow time frame that they are outside of the berry, this pest is extremely difficult to control using pesticides alone [1,9]. Most coffee-growing regions are transitioning to an Integrated Pest Management (IPM) approach to control CBB. IPM typically involves a combination of sanitation practices (e.g., pruning and strip picking), biological controls (e.g., predatory beetles or parasitoid wasps), and the application of biopesticides (e.g., the entomopathogenic fungus *Beauveria bassiana*) [20,21,22,23,24]. Despite a good environmental profile and effectiveness against a wide variety of crop pests such as codling moth [25,26], tephritid flies [27,28,29], and brown-marmorated stinkbug [30,31], exclusion systems have not been examined as a potential IPM strategy for the protection of coffee crops against CBB.

Exclusion nets have been used in agriculture since the mid-20th century as a pest control tool [32]. These systems act as a physical barrier to deny access to the crop and can be grouped into two categories: complete or incomplete exclusion [33]. Complete exclusion refers to row-by-row systems in which the soil is also excluded from the zone, whereas incomplete exclusion refers to full block netting systems covering entire orchards [34] or row-by-row systems in which the nets are left hanging down to the ground [35]. Exclusion nets are almost exclusively made of high-density polyethylene (HDPE) and have an average lifespan of 5–15 years under field conditions [31,33]. 

Exclusion nets have long been considered un-economical to use, despite their sustainability [35] and efficiency under variable environmental conditions [33,36]. This view has gradually changed over the last two decades, as the search for sustainable alternatives to chemical controls is being increasingly driven by pesticide bans and public demand for organically grown food [37,38]. A growing body of research in recent years has shown that exclusion nets are effective in preventing or significantly reducing crop damage from various pests including insects [26,27,28,39,40,41,42], birds [43,44,45] and bats [46], as well as weather-related factors such as wind [47] and hail [48]. Additionally, exclusion nets modify the plant’s growing environment and can have a number of positive effects on the physiology of the plant (e.g., reducing water stress by lowering evaporative demand, reducing heat stress by shading, and reducing light stress by improving the penetration of spectrally modified light into the canopy) [49,50,51,52]. This reduction in environmental stressors often translates to improved photosynthesis and vegetative development, increased fruit set, larger fruit size, changes in time to maturation (either advancing or delaying harvest), and improved fruit quality [53,54]. Crops for which exclusion systems are best suited are those that are high value, perennial, and grown over a logistically manageable space for net installation. Exclusion systems are now widely used for a range of agricultural commodity crops including raspberries, blueberries, blackberries, grapes, nectarines and apples in many countries around the world. 

CBB was first detected on Hawai’i Island in 2010 in the Kona coffee-growing district [55], an area that is known worldwide for its high-quality coffee [56]. CBB quickly spread to the ~800 coffee farms on the island (mostly small, family-owned operations, ~0.5–1 ha in size, that employ manual harvesting), and was later detected on the neighboring islands of Oʻahu (2014) and Maui (2016), which have primarily large estates that employ mechanical harvesting. Hamilton et al. [57] reported 2–3 CBB generations per season at high elevation farms on Hawai’i Island, while low elevation farms had 4–5 generations per season. Poorly managed farms can have infestation levels in excess of 60% [58], resulting in huge economic losses. The estimated economy-wide impact due to CBB in Hawaii is ~$21M annually [59]. While many growers in Hawaii have adopted IPM strategies to manage CBB populations, including monthly sprays of *Beauveria bassiana* and an end-of-season strip picking of all coffee fruits, these practices are both costly and labor intensive [22,23]. Hawaii’s coffee industry is currently experiencing a major labor shortage, resulting in many growers being unable to maintain the high level of sanitation necessary to limit CBB reproduction cycles. 

The factors that characterize Hawaii’s coffee industry (high crop value, limited physical extent, and high labor and production costs) make exclusion nets a potentially viable option for managing CBB and improving crop quality in the islands. The aim of this study was to explore the efficacy, feasibility, and limitations of using exclusion nets to control CBB in coffee farms in Hawaii. Specifically, we examined the effect of exclusion netting on CBB infestation, population per fruit, sex ratio, CBB mortality by *B. bassiana*, crop yield, and coffee quality. We also measured temperature, relative humidity and solar radiation in exclusion plots and un-netted control plots to see how exclusion netting affected the microclimate.

## 2. Materials and Methods

### 2.1. Study Sites

The exclusion was implemented from May–December 2018 at two commercial coffee farms located at 279 and 393 m elevation in the Ka’u district of Hawai’i Island. These farms are part of an ongoing, area-wide CBB monitoring program conducted by USDA-ARS, and were selected for the study based on two criteria: 1) consistently high CBB infestation (≥20%) at the end of the 2016 and 2017 growing seasons, and 2) minimal potential for exclusion nets to interfere with management (i.e., mowing or spraying). Management strategies at both farms consisted of pruning, fertilizer applications, weed management, 7–10 applications of *B. bassiana*, regular fruit harvesting, and end-of-season strip picking. Farm 1 (279 m) grew *Coffea arabica* var. *catuai* primarily in full sun with a few scattered monkeypod (*Samanea saman*) trees, while Farm 2 (393 m) grew *Coffea arabica* var. *typica* in full sun. 

### 2.2. Experimental Design

Laboratory trials were first conducted using several types of exclusion material to determine the appropriate mesh size needed for the study. Twenty adult female CBB were placed in each of four plastic cups, and each of the cup openings were covered with one of the four possible types of exclusion mesh. Each cup was placed upside down over a second plastic cup that contained green and ripe coffee fruits. The number of CBB that successfully cleared the mesh exclusion material to reach the coffee fruits was counted after 48 h. Based on the results of this trial, we selected the OptiNet 50 Mesh insect netting (mesh size = 50 holes per square inch; Green-Tek, Visalia, CA, USA), which prohibited CBB from passing through and reaching the coffee fruits. 

**Figure 1:**
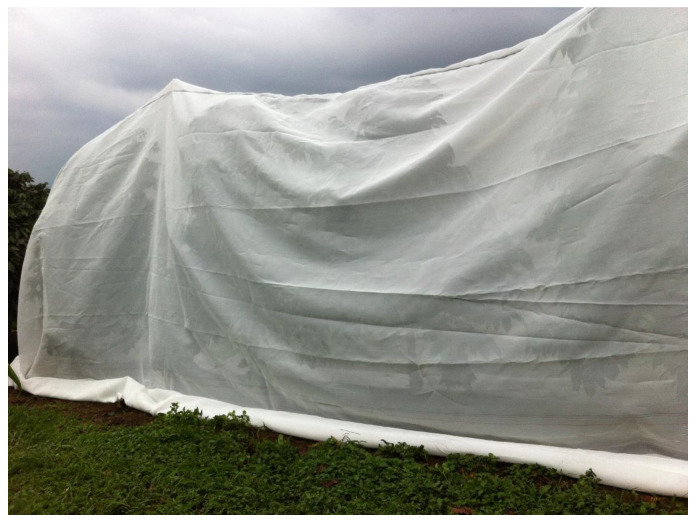
Photograph of exclusion plot containing five trees covered with fine-mesh netting. Four replicate exclusion plots were positioned randomly throughout each of the two farms and were paired with adjacent control (un-netted) plots.

Prior to the start of the study, workers conducted post-harvest sanitation on both farms by strip picking the remaining fruits and raisins from all the coffee trees in the entire field (December), followed by annual pruning (January). A randomized complete block design was used at both farms. Four blocks of 10 trees were randomly distributed throughout a 1 ha plot, with each block comprising two adjacent rows of five contiguous trees. Each of the two treatments (control or exclusion) were randomly assigned to a row of five trees within each block (N = 20 trees per treatment/farm). After the major flowering period (February–April), only the trees within control and exclusion plots were subjected to a second round of sanitation picking to remove all infested fruits and raisins. All fruits/raisins were also removed from the ground in control and exclusion plots using a garden hoe in order to limit starting infestation. Immediately after sanitation, nets were draped over the trees receiving the exclusion treatment (May 2018). An incomplete exclusion design was used (i.e., the ground was not excluded), with nets extending to the ground and the edges secured with sandbags (Figure 1). Each net was elevated above the tops of coffee trees using 6-ft T-posts that were driven 2.5 ft into the ground (one on either end of the five-tree plot), and then covered with a 10-ft PVC pipe (see Figure 1). Access into each exclusion plot was through a zippered entryway at one end of the net. The adjacent paired control trees in each block were left un-netted. Farm management practices were not altered in any way during the experiment; growers used a tractor sprayer to conduct regular (~monthly) applications of BotaniGard^®^ ES (including applications to control and exclusion plots), which is a commercial formulation of *Beauveria bassiana* commonly used in Hawaii to control CBB. Foliar fertilizer was occasionally added to the spray mixture. Weeds were controlled using herbicide sprays and manual control. Workers were instructed not to pick coffee from experimental blocks, which were clearly demarcated with signs and temporary fencing. 

### 2.3. Microclimate

To estimate the effect of exclusion netting on microclimate, we monitored temperature, relative humidity (RH), and solar radiation within two exclusion plots and two control plots on each farm. Temperature and RH was measured using HOBO Pro v2 data loggers (U23-002, Onset Computer Corporation, Bourne, MA, USA) placed in solar shields (RS3, Onset Computer Corporation, Bourne, MA, USA). Solar radiation was measured using solar pendants (UA-002-64, Onset Computer Corporation, Bourne, MA, USA). Loggers were placed on 4-ft posts at the center of each plot, and measurements were logged every hour. For each farm, daily averages of the mean, maximum, and minimum for each variable were calculated and averaged across the two loggers in each of the control and exclusion treatments. 

### 2.4. CBB Infestation, Position, Population per Fruit, Sex Ratio, and Mortality

Data on CBB infestation were collected bi-weekly in exclusion and control plots for each farm. Percent CBB infestation was estimated for each treatment by examining one randomly selected branch at chest height on each tree following a modified protocol from Johnson et al. [60]. The total number of green fruits and the total number of green infested fruits (based on the presence of an entrance hole in the central disc) was counted for each branch. Once fruits began to ripen, they were collected, separated by treatment, and processed on a bi-weekly basis for the remainder of the season, with harvested fruits separated by treatment. Percent CBB infestation was also estimated in ripe fruits by randomly selecting 300 harvested fruits from each treatment/sampling date and counting the number of infested fruits. From September–December, 25 ripe, infested fruits from each treatment/sampling date were randomly selected and dissected under a dissecting microscope at 30–50× (Leica, Microsystems GmbH, Wetzlar, Germany). To determine if exclusion nets indirectly affected (through changes in microclimate) the rate at which CBB entered the fruit and commenced reproduction, we recorded the position of the founding female CBB within the fruit (AB: founding female has commenced boring into the fruit but has not entered the endosperm, or CD: founding female has entered the endosperm and is either in the process of building galleries for reproduction or has already produced offspring). We also recorded the total number of individuals per fruit in each of the five major life stages (eggs, larvae, pupae, teneral adult, and mature adult) and the sex ratio of adult individuals (teneral and mature) to investigate if development and sex determination were affected by potential differences in temperature between treatments. Lastly, the mortality rate of adults due to *B. bassiana* infection (as evidenced by white mycelial growth protruding from dead CBB) was recorded to determine if nets had any impacts on the effectiveness of this biopesticide.

### 2.5. Coffee Fruit Size, Yield and Quality

Weight (g) and diameter (mm) was measured for 25 randomly selected un-infested ripe fruits for each treatment/sampling date to estimate fruit size. The weight of all harvested fruits for each treatment/sampling date was then recorded, and the total weight for each treatment/farm was later divided by the number of trees per treatment to get an average yield per tree (kg) for the season. Harvested coffee was stored at 10 °C then sorted and processed (within 1–2 days) at the USDA-ARS DKI US Pacific Basin Agricultural Research Center (PBARC) to be used for coffee sensory evaluations as a measure of quality. Sorting was done by visual inspection to ensure optimal and uniform ripeness for quality assessments. Ripe fruits selected by manual sorting were then floated in water for density evaluation, and any floating fruits were removed from further processing. The sorted fruits were pulped using a manual hand-crank pulper (DH-2, Penagos, Bucaramanga, Colombia), and further sorting was conducted after pulping to remove any beans exhibiting damage from the pulper, insects, fungus, or microbes. The coffee samples from each of the two treatments were fermented in 2 L plastic containers covered with fine-mesh lids. For each trial, 1000 g of pulped coffee was submerged in 400 g of tap water and mixed every 4–8 h. Fermentation was carried out at room temperature (21–23 °C) for 24 h. 

Following fermentation, beans were rinsed in a strainer until all mucilage was removed from the parchment, and were distributed in a single layer on wire-mesh drying racks and placed in a sealed room set to a temperature of 21–23 °C and a RH of 65–75% for a period of 5–7 days. The coffee beans were turned hourly for the first four hours and then twice per day for the remainder of the drying period. After the first stage of drying, beans were moved to a room with temperatures between 21–27 °C and RH 65–75%. Fans were set up to ensure constant airflow and to assist drying. When samples reached ~17–20% moisture content, they were moved to environmental chambers at a temperature of 26 °C and RH of 50%. Beans were turned twice per day and tested periodically for moisture content with a coffee moisture meter (Shore Measuring Systems, Attica, IN, USA) over a period of 2–4 days. Beans were removed from the chamber when they reached a parchment moisture content of 10.5%. At the completion of drying all samples were stored at 10 °C in labeled Ziploc bags, which were placed in a hermetic bag (GrainPro, Concord, MA, USA) and sealed in a 5-gallon bucket. Samples were stored for a minimum of 1.5 months prior to cupping evaluations. 

The parchment was then removed using a standard coffee huller. Coffee samples were hulled at starting temperatures of 10 °C, and hulling times were kept below one minute to ensure the coffee did not heat up excessively. After hulling, the green beans were visually sorted to remove all defective beans from samples. Fifty-gram samples from each treatment and harvest date were prepared for roasting from each farm. All sample roasts for cupping evaluations were performed on a professional roaster (Ikawa Pro, Ikawa, London, UK) to Specialty Coffee Association (SCA) Agtron “Gourmet” standards using a colorimeter (CM-100, Lighttells, Zhubei, Taiwan). Following the completion of the roast and cooling phase, coffee samples were stored in non-permeable bags at room temperature until they were cupped 24 h later to minimize exposure to air and prevent contamination. 

Each roasted coffee sample was separated into five 8.25 g samples for SCA sensory evaluation. Samples were ground on a commercial coffee grinder (EK43 S, Mahlkönig, Germany) less than 15 min prior to the onset of fragrance evaluation. Grind particle size conformed with SCA requirements of 70–75% pass through on a U.S. standard size 20-mesh sieve. Mineral-enhanced flavor optimizing coffee brewing water (Third Wave Water, Columbus, OH, USA) was used for all cupping evaluations. Water was heated to 96 °C, and 150 g of water was poured into all evaluated samples per the SCA Cupping Protocol guidelines. Crust break started at four minutes, and sample evaluation ended once coffees reached room temperature (21 °C). 

Coffee samples from four separate trials per farm (harvested from September–December 2018) were evaluated by five industry professionals. Two of the panel members were certified Q-graders (capable of analyzing arabica coffee through smell and taste as determined by the Coffee Quality Institute), and the remaining members were selected industry professionals who have demonstrated proficiency scoring and evaluating Hawaiian coffees via participation in Hawaiian regional and state competition events, as well as a range of Hawaiian coffee consulting and industry applications related to coffee quality. All coffee samples were numerically coded and cupped without participant awareness of the farm, harvest, or treatment. Samples were scored on the SCA 100-point system, and included assessments of fragrance/aroma, flavor, aftertaste, acidity, mouthfeel, and balance. Coffee evaluators filled out their scores independently and submitted them for data entry. 

### 2.6. Statistical Analysis

Each dataset was first examined with individual boxplots to detect outliers (data points that fall more than 1.5 times the interquartile range above the third quartile or below the first quartile); only a single outlier was identified and subsequently removed. Normality was then assessed by plotting histograms and conducting the Shapiro–Wilk test, followed by an *F*-test to assess the data for equal variances. Proportion data were arcsine transformed prior to analysis. Given that the assumptions of normality and equal variances were not met, non-parametric statistics were used. Control and exclusion plots were compared for each variable using paired Wilcoxon signed-rank tests. All analyses were conducted in R v. 3.5.0 using the ‘stats’ package [61].

## 3. Results

### 3.1. Microclimate

The mean daily temperature inside exclusion plots was nearly identical to that in un-netted control plots at ~22 °C for both farms (Table 1). The mean daily range in temperature was ~1 °C higher in exclusion plots relative to control plots for both farms (Table 1). Daily mean RH was 0.4–2% lower in exclusion plots on both farms, while the mean daily range in RH was ~4% higher in exclusion plots relative to control plots (Table 1). The daily mean solar radiation was approximately twice as high in control plots compared to exclusion plots; the same trend was observed for the daily maximum solar radiation (Table 1). 

### 3.2. CBB Infestation and Position

**Figure 2:**
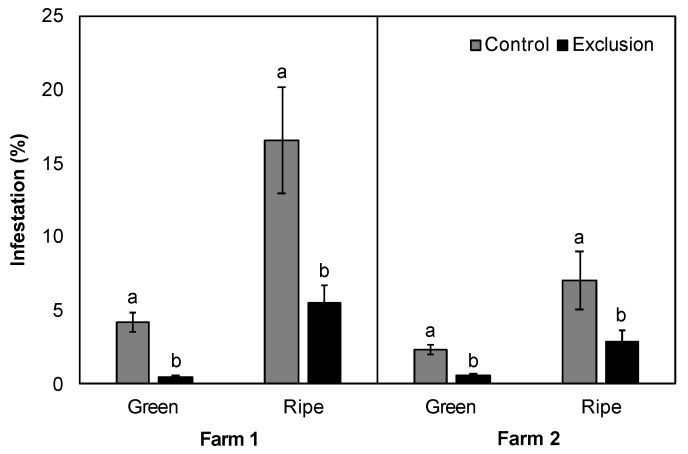
Percent (mean ± SE) CBB infestation in green and ripe coffee fruit sampled from control (un-netted) and exclusion plots across two coffee farms on Hawai’i Island. Lowercase letters represent significant differences in the means.

The mean percentage of infested green fruits (Farm 1: V = 78, *p* < 0.001; Farm 2: V = 63, *p* = 0.005; Figure 2) and infested ripe fruits (Farm 1: V = 74, *p* = 0.007; Farm 2: V = 70, *p* = 0.02; Figure 2) was significantly higher in the control plots relative to exclusion plots on both farms. Infestation of green fruits in control plots ranged from 1–9%, compared to 0–2% infestation in exclusion plots for the duration of the season (Figure S1). The infestation of ripe fruits from exclusion plots showed a decreasing trend throughout the harvest season (Figure S1), while the infestation of ripe fruits from control plots was more variable (range = 1–39%) with peaks early (July–August) and late (November–December) in the harvest season (Figure S1). The proportion of CBB in the AB position (Farm 1: V = 5, *p* = 0.59; Farm 2: V = 21, *p* = 0.27) and CD position (Farm 1: V = 10, *p* = 0.59; Farm 2: V = 8, *p* = 0.34) was not significantly different in control and exclusion plots throughout the sampling period at both sites (Figure S2).

### 3.3. CBB Population per Fruit, Sex Ratio and Mortality

**Figure 3:**
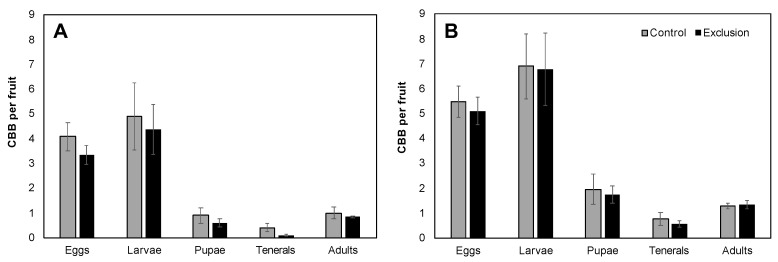
Mean (± SE) number of CBB per ripe fruit separated into the main life stages (eggs, larvae, pupae, teneral adults and mature adults) for Farm 1 (**A**) and Farm 2 (**B**). There was no significant difference between control and exclusion plots in the mean number of CBB per fruit in any of the five life stages examined (*p* > 0.05).

**Figure 4:**
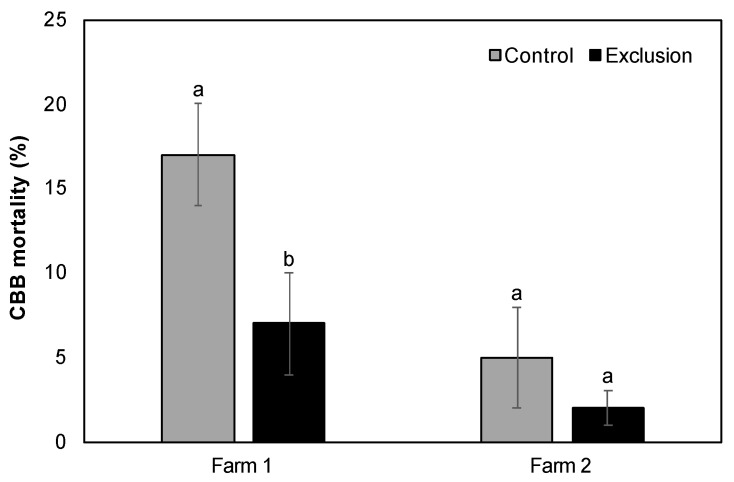
Percent CBB mortality (mean ± SE) from *Beauveria bassiana* in ripe fruits on two Hawai’i Island farms. Lowercase letters represent significant differences in the means.

The mean number of CBB per infested fruit across the entire sampling period was not significantly different between control and exclusion plots (Farm 1: 11.27 ± 2.05 vs. 9.21 ± 1.04, V = 13, *p* = 0.16; Farm 2: 16.32 ± 2.02 vs. 15.49 ± 2.03, V = 14, *p* = 1.00). Although the observed decrease in each of the five life stages in exclusion plots relative to control plots was not significant for either farm (*p* > 0.05; Figure 3), when considering the cumulative CBB load over the entire season, exclusion plots saw a 7–18% reduction in eggs, 2–11% reduction in larvae, 11–44% reduction in pupae, 25–78% reduction in teneral adults, and 0–16% reduction in mature adults compared to control plots. We also observed a non-significant decrease in the mean ratio of adult females to males in exclusion plots relative to control plots (Farm 1: 8:1 vs. 10:1, V = 9, *p* = 0.80; Farm 2: 12:1 vs. 14:1, V = 31, *p* = 0.48). Lastly, the mean percentage of adult CBB with evidence of mortality by *B. bassiana* in ripe fruits was significantly higher in control relative to exclusion plots for Farm 1 (V = 21, *p* = 0.03; Figure 4), but was not different for Farm 2 (V = 9, *p* = 0.27; Figure 4). 

### 3.4. Coffee Maturation, Yield and Quality

**Figure 5:**
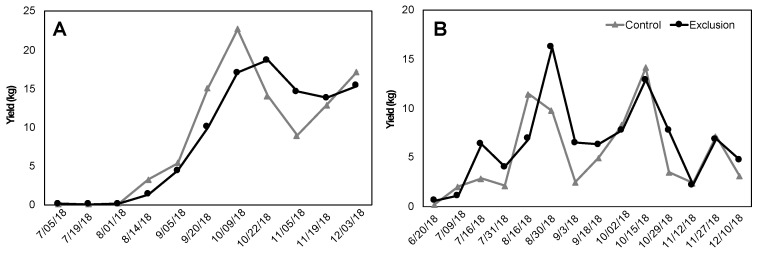
Total yield (kg) of ripe coffee fruit harvested from 20 trees per treatment (un-netted control and netted exclusion) on two Hawai’i Island farms: Farm 1 (279 m, var. *catuai*) (**A**) and Farm 2 (393 m, var. *typica*) (**B**).

**Figure 6:**
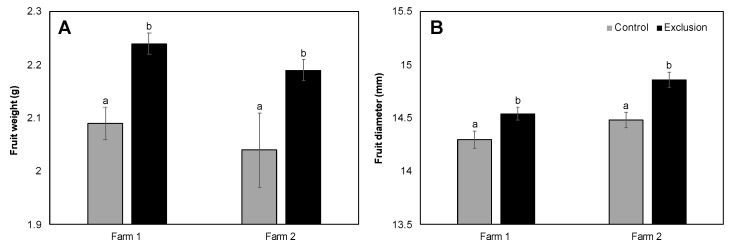
Mean (± SE) weight (**A**) and diameter (**B**) of ripe coffee fruits harvested from 20 trees per treatment (un-netted control and netted exclusion) at two farms on Hawai’i Island (Farm 1: 279 m, var. *catuai*, Farm 2: 393 m, var. *typica*). Lowercase letters represent significant differences in the means.

Fruit maturation times were similar between treatments, with peaks in harvest largely overlapping (Figure 5A,B). Farm 1 (279 m, var. *catuai*) produced similar average yields per tree in exclusion and control plots (4.79 vs. 5.00 kg/tree, V = 42, *p* = 0.48; Figure 5A). Farm 2 (393 m, var. *typica*) produced higher average yields per tree in exclusion plots compared to un-netted controls, but this difference was not significant (4.51 vs. 3.71 kg/tree, V = 31, *p* = 0.19; Figure 5B). Ripe fruits from exclusion plots were on average significantly heavier (Farm 1: V = 112, *p* = 0.002; Farm 2: V = 155, *p* < 0.001; Figure 6A) and had significantly larger diameter than fruits from control plots (Farm 1: V = 119, *p* = 0.004; Farm 2: V = 40, *p* = 0.04; Figure 6B). Lastly, coffee quality for both farms was not significantly different between the two treatments based on the mean cupping score from five separate harvests throughout the season (Farm 1: Control = 82.19 ± 0.27 vs. Exclusion = 82.21 ± 0.20, V = 92, *p* = 0.92; Farm 2: Control = 82.66 ± 0.21 vs. Exclusion = 82.59 ± 0.20, V = 99, *p* = 0.57).

## 4. Discussion

We examined the efficacy and feasibility of using exclusion netting to manage coffee berry borer on two small-scale commercial coffee farms in the Ka’u coffee-growing district of Hawai’i Island. Our results suggest that netting can significantly reduce infestation by CBB without negatively impacting coffee yield or quality. Across the sampling period from June–December at both farms, we observed significantly lower infestation in both green and ripe fruits. Although we observed a reduction in the number of CBB per fruit in all life stages and a reduction in the ratio of female:male adults per fruit in exclusion plots, these decreases were not significant. CBB mortality by the entomopathogenic fungus *B. bassiana* was significantly higher in control plots for one of the two farms. We observed significantly larger fruits in exclusion plots, but average yields per tree were not different between treatments. Lastly, no difference was found between exclusion and control plots in the average coffee quality based on cupping scores.

Through the use of exclusion netting we observed an average decrease of 7.59% (range = 0.33–35%) in ripe fruit infestation across both farms. Importantly, coffee growers on Hawai’i Island must meet the threshold of <10% CBB infestation in order to receive top price for coffee cherry when selling to major processors, and harvests with over 25% CBB damage may be rejected. Of the 11–12 rounds of harvesting conducted on each farm, 3–4 rounds had infestation levels above 10% in control plots (compared to 1–2 rounds in exclusion plots) and 2 rounds had infestation levels above 25% in control plots (compared to zero rounds in exclusion plots). The majority of infestation in control plots can be attributed to CBB migration from neighboring trees/ground areas. While all trees on both farms were strip-picked prior to the start of the season, it is likely that some infested fruits/raisins were missed. According to Aristizábal et al. [23], a survey of harvesting efficiency on 11 Hawai’i Island coffee farms found that 70% of harvesting rounds could be rated as ‘bad’, having >10 fruits/tree remaining. These remaining tree raisins have been shown to harbor an average of 20 CBB/raisin on Hawai’i Island farms [62]. Additionally, ground raisins from below neighboring trees were not removed due to labor costs, and these have been shown to harbor an average of 5 CBB/raisin [62]. In contrast to the control plots, CBB from neighboring tree/ground areas were likely prohibited from entering exclusion plots due to the exclusion net’s optical additives (which repel insects) and fine-mesh size (which physically exclude insects). Therefore, any infestation observed in exclusion plots was likely due to residual CBB populations that emerged from tree and/or ground raisins that were missed when plots were cleared prior to installing nets. This was reflected by the fact that the highest levels of infestation observed in exclusion plots were in the first two rounds of harvest, after which infestation levels fell sharply. This suggests that thorough pre-installation sanitation is crucial to the success of any exclusion system for CBB. However, if some CBB persist after nets are erected the population can be effectively reduced by frequent harvesting.

Although we observed significantly lower infestation in exclusion plots, we did not find significant differences in CBB populations or sex ratios per fruit (typically skewed 10:1 towards females). Mariño et al. [63] reported a decrease in the number of eggs, larvae, pupae and adults in shade plots relative to plots in full sun, as well as a lower female:male CBB ratio in shade compared to sun. The authors suggested that decreased temperatures in shade likely slowed generation time, and also favored the development of males relative to females. In contrast to the results of this study, mean daily temperatures and mean ranges in daily temperatures were highly similar between exclusion and control plots, which likely explains why we did not find a significant difference in CBB populations or sex ratios between treatments. Differences between the results of these studies are likely tied to differences in percent shade (79% vs. 50%), as well as the fact that our shaded plots were enclosed by netting which likely decreased air flow, thereby increasing temperatures. Lastly, CBB mortality due to *B. bassiana* was higher in control plots relative to exclusion plots (significantly so for one of the two farms). The decrease in mortality observed in exclusion plots may have been caused by a lower penetration rate of *B. bassiana* through the fine-mesh exclusion nets during applications. A reduced ability of spray applications to penetrate through nets (for purposes of foliar fertilization and supplemental pesticides) must be considered as a possible limitation of using exclusion netting. However, these could be sprayed directly on trees under exclusion nets using backpack sprayers (for small exclusion systems) or tractor sprayers (for large exclusion systems). 

Exclusion systems did not greatly alter temperature or RH. We observed slightly higher mean daily temperatures and slightly lower mean daily RH in exclusion plots compared to un-netted control plots. In contrast, solar radiation in exclusion plots was ~50% lower compared to that in control plots. This result was in accordance with the manufacturer’s specifications for the netting (50–52% shade). However, the large decrease in solar radiation recorded in exclusion plots did not negatively impact coffee yield or quality. In fact, we observed that fruits harvested from exclusion plots were larger on average than those from control plots. Increases in fruit size and weight may be related to the slight increase in daily temperatures observed in exclusion plots, as was also reported in blueberries [64] and raspberries [28]. Additional research is needed to determine if the trend reported here for larger, heavier fruits under exclusion nets holds when examined across a larger sample of coffee farms at varying elevations.

The most commonly grown variety of coffee in Hawaii, var. *typica*, showed higher average yields per tree in exclusion plots compared to un-netted controls, although this difference was not significant. Yields from var. *catuai* were slightly lower in exclusion plots relative to control plots but were also not significantly different. In a five-year exclusion study on apples, Chouinard et al. [54] reported on average larger fruits and lower fruit load in exclusion plots compared to un-netted control plots, but equivalent total yields between treatments across all years. The authors suggested that differences in fruit size and load between treatments might have been more influenced by pollination (lower numbers of seeds per fruit in exclusion plots due to decreased insect pollination) than the exclusion nets themselves and their resulting influence on microclimate conditions. In the present study, we erected exclusion nets in May following the peak in flowering (March–April). Flowers were occasionally observed after this period, and it is possible that without insect pollination many of the resulting fruits from sporadic flowerings were peaberries (only one of the two ovules was fertilized, producing a single seed per fruit), although we did not follow this specifically. One possible way to increase fruit set, fruit weight, and yield while using exclusion systems in coffee would be to place beehives within larger exclusion blocks. In Hawaii, many beekeepers have a mutual agreement with coffee growers that allows them to place hives around their farms during the flowering season. The bees provide pollination services to coffee growers, while increasing their production of honey and other bee products to be sold commercially. Further studies are needed to explore optimal installation designs that would permit the inclusion of beehives in exclusion systems. Research is also needed to determine if yields can be increased by using exclusion netting with lower shade levels (<50%). 

While the present study found exclusion netting to significantly reduce CBB infestation in two small-scale coffee farms, there are several potential concerns that need to be addressed before growers can consider including exclusion systems as a practical part of an IPM plan for CBB control. First, the cost of the netting itself, and the cost of labor to install and maintain the exclusion system must be considered. Installation of row-by-row systems as was done in the present study will require little in the way of net support, but may impose difficulties for management activities and crop harvest. More long-term installation designs that would make these activities easier to perform will require structural support for the nets, adding to the initial investment cost for labor and materials. Recent studies using exclusion netting to protect against insect pests in raspberries [28] and grapes [29] estimated a cost of 6000 USD/acre (including cost of netting, shipping, support systems, accessories to secure nets, labor, and supplemental pollination). The amortized cost of these exclusion systems would then be 1200 USD/acre based on a minimum net lifespan of five years, as is projected for the netting used in the current study. Detailed economic analyses are needed to determine the actual cost per acre for exclusion systems in coffee, and if the reduction in pesticide applications and potential increase in fruit quality and yield (in the form of larger, heavier fruits and higher numbers of marketable beans) would be sufficient to cover the initial investment costs. One way for growers to offset the initial investment costs would be to market coffee grown under exclusion nets as organic, sustainable, and shade-grown, thereby increasing the value of their coffee products on the world specialty market. A second potential concern that will need to be further investigated is how exclusion netting affects other coffee pests and diseases (e.g., aphids, sooty mold, leaf rust, etc.), as well as beneficial insects. In the present study, we did not observe any obvious increase in pest/fungal populations under exclusion netting, but additional studies are needed to monitor these potential issues over multiple years and across a range of environmental conditions. 

## 5. Conclusions

The findings presented here are an important first step in exploring physical barriers for the protection of coffee from CBB as part of a comprehensive IPM program. Pesticide resistance, increased demand for organically grown products, and problems related to climate change all present significant challenges for traditional pest control. Innovative and sustainable options for overcoming these challenges are greatly needed, particularly for regions that have high production costs and labor shortages (e.g., Hawaii and Puerto Rico). For small-scale growers, exclusion systems have the potential to improve fruit quality, while saving time and money by eliminating the need to apply costly pesticides [65,66]. Furthermore, nets can provide a level of assurance to growers that they will have a marketable crop even if labor is insufficient to conduct sanitation and frequent harvesting [65,66]. The initial cost of installation can be offset in that many exclusion nets currently available are of high-quality material that can be re-used for 5–15 years [31,33,65], and coffee grown sustainably under nets can be marketed as organic and shade-grown to increase value on the world specialty market. Future research will explore varying themes on the exclusion system described here for coffee, including studies seeking to optimize the timing and height of net placement, and the most cost-effective materials and installation designs. Additional research is planned to examine the use of border crops that could act as a physical barrier to CBB movement while providing income to the grower. Two examples of potential border crops that could work well in tropical coffee-growing regions are Sunn hemp (*Crotolarea juncea*) and Sorghum-Sudangrass hybrids (*Sorghum bicolor* × *Sorghum bicolor* var. *sudanese*), both of which provide biomass to be sold as livestock feed, erosion control, soil improvement, and suppression of weeds and root-knot nematode [67,68]. 

## Figures and Tables

**Table 1:** Mean (± SE) daily values (mean, maximum, and minimum) for three microclimate variables (temperature, relative humidity, and solar radiation) averaged across two replicate sensors per treatment at two sites on Hawai’i Island from July–December 2018.

Microclimate Variable	Farm 1		Farm 2	
	Control	Exclusion	Control	Exclusion
Mean Temp (°C)	22.47 ± 0.10	22.50 ± 0.10	22.11 ± 0.09	22.46 ± 0.09
Max Temp (°C)	28.29 ± 0.14	29.36 ± 0.16	27.62 ± 0.13	29.03 ± 0.17
Min Temp (°C)	17.90 ± 0.15	17.50 ± 0.15	17.68 ± 0.14	18.02 ± 0.12
Mean RH (%)	84.30 ± 0.61	83.92 ± 0.59	84.35 ± 0.59	82.32 ± 0.58
Max RH (%)	96.04 ± 0.54	96.60 ± 0.54	95.58 ± 0.53	94.81 ± 0.53
Min RH (%)	64.91 ± 0.76	61.78 ± 0.73	64.01 ± 0.77	59.39 ± 0.75
Mean Solar (Lux)	7212.78 ± 525	4430.95 ± 290	23,196.38 ± 884	11,311.71 ± 532
Max Solar (Lux)	43,075.87 ± 3064	27,304.94 ± 1818	142,875.07 ± 5130	65,473.95 ± 2867

## Supplementary Materials

The following are available online at https://www.mdpi.com/2075-4450/11/6/364/s1, Figure S1: Ripe fruit infestation over time in two Hawai’i Island coffee farms, Figure S2: Position of CBB (AB or CD) within coffee fruits on two Hawai’i Island coffee farms.
