# Supplementary material for: Evaluation of Exclusion Netting for Coffee Berry Borer (Hypothenemus Hampei) Management
Source: Insects. 2020 Jun 11;11(6):364. doi: 10.3390/insects11060364 (PMC7348803; doi:10.3390/insects11060364)
Supplement: Supplementary file 1 [file insects-11-00364-s001.pdf]

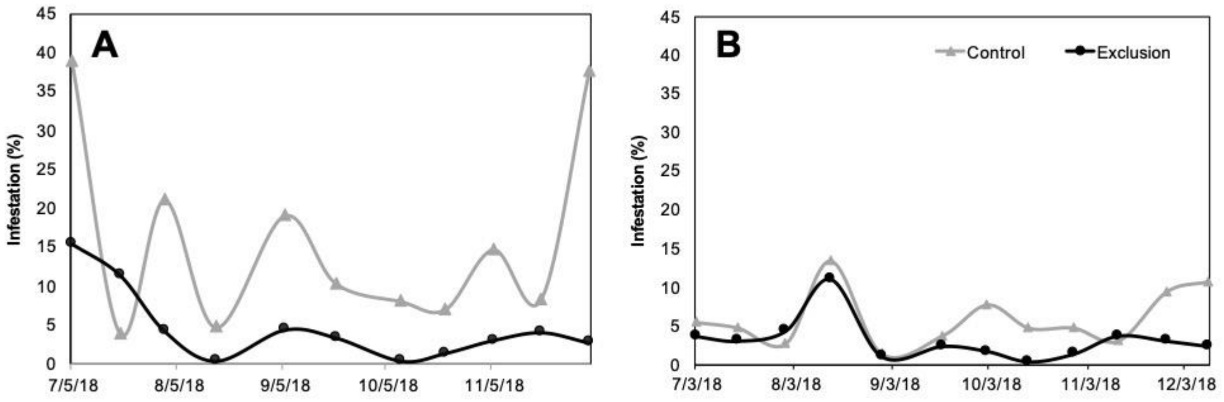

Figure S1. CBB infestation of ripe coffee fruits over time in control (un-netted) and exclusion treatments on two Hawai'i Island coffee farms: Farm 1 is located at 279 m elevation and grows var. *catuai* (A), while Farm 2 is located at 393 m elevation and grows var. *typica* (B).

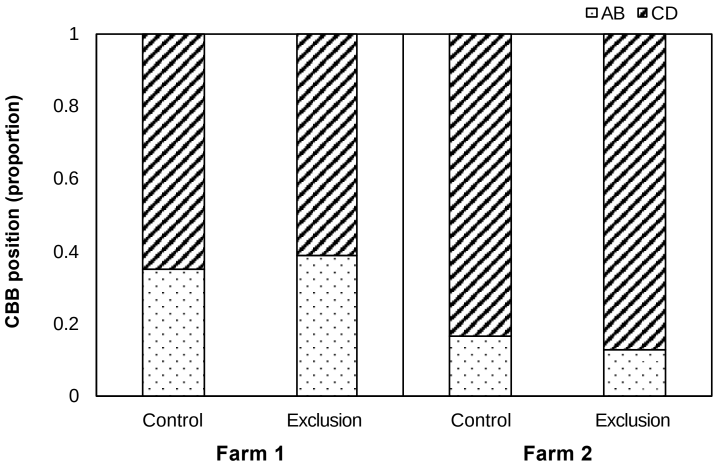

Figure S2. Position of adult founding female CBB within coffee fruit collected from control (un-netted) and exclusion plots at two Hawai'i Island coffee farms. The AB position indicates the female has initiated boring into the fruit but has not reached the endosperm, while position CD indicates the female has entered the endosperm and is either in the process of building galleries for reproduction or has already produced offspring.
